# Antigenic Change in Human Influenza A(H2N2) Viruses Detected by Using Human Plasma from Aged and Younger Adult Individuals

**DOI:** 10.3390/v11110978

**Published:** 2019-10-23

**Authors:** Yukimasa Matsuzawa, Kiyoko Iwatsuki-Horimoto, Yoshinori Nishimoto, Yukiko Abe, Satoshi Fukuyama, Taiki Hamabata, Moe Okuda, Yui Go, Tokiko Watanabe, Masaki Imai, Yasumichi Arai, Ron A.M. Fouchier, Seiya Yamayoshi, Yoshihiro Kawaoka

**Affiliations:** 1 Division of Virology, Department of Microbiology and Immunology, Institute of Medical Science, University of Tokyo, Minato-ku, Tokyo 108-8639, Japan; ymatsuza@ims.u-tokyo.ac.jp (Y.M.); fukuyamarry@gmail.com (S.F.); kk176356@stu-cbms.k.u-tokyo.ac.jp (M.O.); yuigo555@gmail.com (Y.G.); tokiko.watanabe@wisc.edu (T.W.); mimai@ims.u-tokyo.ac.jp (M.I.); yamayo@ims.u-tokyo.ac.jp (S.Y.); 2 Center for Supercentenarian Medical Research, Shinjuku-ku, Tokyo 160-8582, Japan; ynishimo@z3.keio.jp (Y.N.); yukiko_abe@keio.jp (Y.A.); yasumich@keio.jp (Y.A.); 3 Department of Viroscience, Erasmus Medical Center, Doctor Molewaterplein 40, 3015 GD Rotterdam, The Netherlands; r.fouchier@erasmusmc.nl; 4 Department of Special Pathogens, International Research Center for Infectious Diseases, Institute Medical Science, University of Tokyo, Minato-ku, Tokyo 108-8639, Japan; 5 Influenza Research Institute, Department of Pathobiological Sciences, School of Veterinary Medicine, University of Wisconsin-Madison, Madison, WI 53706, USA

**Keywords:** influenza A virus, aged individuals, H2N2, antigenic drift, antigenic change

## Abstract

Human influenza A(H2N2) viruses emerged in 1957 and were replaced by A(H3N2) viruses in 1968. The antigenicity of human H2N2 viruses has been tested by using ferret antisera or mouse and human monoclonal antibodies. Here, we examined the antigenicity of human H2N2 viruses by using human plasma samples obtained from 50 aged individuals who were born between 1928 and 1933 and from 33 younger adult individuals who were born after 1962. The aged individuals possessed higher neutralization titers against H2N2 viruses isolated in 1957 and 1963 than those against H2N2 viruses isolated in 1968, whereas the younger adults who were born between 1962 and 1968 possessed higher neutralization titers against H2N2 viruses isolated in 1963 than those against other H2N2 viruses. Antigenic cartography revealed the antigenic changes that occurred in human H2N2 viruses during circulation in humans for 11 years, as detected by ferret antisera. These results show that even though aged individuals were likely exposed to more recent H2N2 viruses that are antigenically distinct from the earlier H2N2 viruses, they did not possess high neutralizing antibody titers to the more recent viruses, suggesting immunological imprinting of these individuals with the first H2N2 viruses they encountered and that this immunological imprinting lasts for over 50 years.

## 1. Introduction

In 1957, an influenza A(H2N2) virus caused the so-called Asian flu. The pandemic H2N2 virus became a seasonal influenza virus and continued to circulate until 1968, when the H3N2 pandemic virus replaced the H2N2 virus [1]. The H2N2 viruses have not circulated in the human population since then. Therefore, most humans who were born after 1968 lack immunity, including neutralizing antibodies, to the H2N2 viruses [2,3].

Between 1957 and 1968, the human H2N2 viruses accumulated amino acid mutations in their HAs to escape from neutralizing antibodies [4] and adapt to humans [5], which may have altered their antigenicity. In fact, ferret antisera against several H2N2 isolates were used to reveal differences in antigenicity among the human H2N2 isolates [6,7,8]. Mouse or human monoclonal antibodies were also used to characterize antigenic changes in the HA [9,10]; however, these experiments used H2N2 isolates that were propagated in embryonated chicken eggs with egg-adapted amino acid mutations in the HA that affect the HA antigenicity. To solve this problem, Linster et al. [11] collected H2N2 isolates that were obtained by culture in tertiary monkey kidney cells and/or MDCK cells for a maximum of five passages, without prior inoculation into embryonated chicken eggs. Using such isolates and ferret antisera against these isolates, they found antigenic changes among H2N2 viruses isolated between 1957 and 1968 [11]. The T128D and N139K substitutions together with the E126T, S154P, A184T, and A188T substitutions around the receptor-binding site of HA were important for the antigenic changes in the H2N2 viruses [11].

In this study, we performed a serological examination of human plasma samples obtained from 50 aged individuals who were born between 1928 and 1933 and 33 younger adult individuals who were born after 1962 to examine how the human immune system sees antigenic changes in human H2N2 viruses.

## 2. Materials and Methods

### 2.1. Ethical Issues

All protocols and experiments in this study were approved by the ethics committees of the Institute of Medical Science at the University of Tokyo (ID: 26-65-1119; date: 7 April 2018) and the Keio University School of Medicine (ID: 20160297; date: 2 April 2018. Written informed consent was obtained from all participants in this study. The Kawasaki Wellbeing Project is registered in the University Hospital Medical Information Network Clinical Trial Registry (ID: UMIN000026053).

### 2.2. Sample Collection

Plasma samples were collected from 50 aged individuals who were born between 1928 and 1933 (85–89 years old) (Table 1). These individuals had participated in the Kawasaki Wellbeing Project, which recruits healthy individuals aged 85–89 years-old, from May to July 2018. Plasma samples from 33 younger adult volunteers born after 1962 (23–55 years old), who participated in a seasonal influenza vaccine surveillance project, were collected before these individuals received the seasonal influenza vaccine from January to November 2018 (Table 1). Information on the vaccination history of most of the individuals was limited. Each blood sample was collected in a 5-mL vacuum tube containing EDTA-2Na (Terumo, Tokyo, Japan). Plasma was separated from whole blood by using a Leucosep (Greiner Bio-One, Kremsmünster, Austria) and was stored at −20 °C until use.

### 2.3. Biosafety Consideration

All experiments with human H2N2 viruses were conducted in a biosafety level (BSL) 3 laboratory at the University of Tokyo, which is approved for such use by the Ministry of Agriculture, Forestry, and Fisheries, Japan.

### 2.4. Cells and Viruses

Madin–Darby canine kidney (MDCK) cells were maintained in Eagle’s minimal essential medium (MEM) containing 5% newborn calf serum at 37 °C in 5% CO_2_. Influenza A(H2N2) viruses, A/Netherlands/M1/57 (M1/57), A/Netherlands/K1/63 (K1/63), A/Netherlands/B1/68 (B1/68), and A/Netherlands/B2/68 (B2/68), were kindly provided by Ron Fouchier (Eramus Medical Center, Rotterdam, Netherlands) [11]. The isolates were obtained by culture in tertiary monkey kidney cells and/or MDCK cells for a maximum of five passages, without prior inoculation into embryonated chicken eggs [11]. The 50% tissue culture infectious dose (TCID_50_) was determined with MDCK cells and the Reed and Muench formula.

### 2.5. Virus Neutralization Assay

Virus neutralization assays were conducted by using the methodology described in the WHO manual on Animal Influenza Diagnosis and Surveillance (http://www.wpro.who.int/emerging_diseases/documents/docs/manualonanimalaidiagnosisandsurveillance.pdf) with some modifications. Briefly, plasma samples were initially treated with a receptor-destroying enzyme (RDE II, DENKA SEIKEN Co., LTD, Tokyo, Japan) to remove non-specific inhibitors. Two-fold serially diluted plasma samples were mixed with 100 TCID_50_ of each isolate. The mixtures were incubated for 30 min at 37 °C and were then inoculated into an MDCK monolayer in a 96-well microplate. Cytopathic effect was observed to detect the presence of viral propagation 3 days after inoculation. The reciprocal number of the minimum dilution of plasma needed to suppress the appearance of CPE was used as the neutralization titer.

### 2.6. Phylogenetic Analysis

The HA sequences of 17 viruses described previously [11] were aligned using the software of ClustalW. The phylogenetic tree was inferred by using the Maximum Likelihood method and Jones-Taylor-Thornton (JTT) matrix-based model [12]. The tree with the highest log likelihood (−1787.57) is shown. The percentage of trees in which the associated taxa clustered together is shown next to the branches. Initial tree(s) for the heuristic search were obtained automatically by applying Neighbor-Join and BioNJ algorithms to a matrix of pairwise distances estimated using a JTT model, and then selecting the topology with the superior log likelihood value. The tree is drawn to scale, with branch lengths measured in the number of substitutions per site. There were 329 positions in the final dataset. Evolutionary analyses were conducted by using the software MEGA X [4]. A/mallard/Netherlands/31/2006 virus is defined as an outgroup.

### 2.7. Statistical Analysis

A non-parametric, repeated measures, one-way ANOVA Friedman’s test followed by a two-stage linear step-up procedure of Benjamini, Krieger, and Yekutieli was performed by using GraphPad Prism 7.04 software. *p* values <0.05 were considered significantly different. No samples were excluded from the analysis.

### 2.8. Antigenic Cartography

The neutralization data were analyzed by using antigenic cartography (https://acmacs-web.antigenic-cartography.org/), which is a method to visualize and increase the resolution of neutralization results, as detailed previously [13]. Plasma samples with no or only one numerical antibody titer were not included because they cannot be placed properly in an antigenic map.

## 3. Results and Discussions

**Figure 1:**
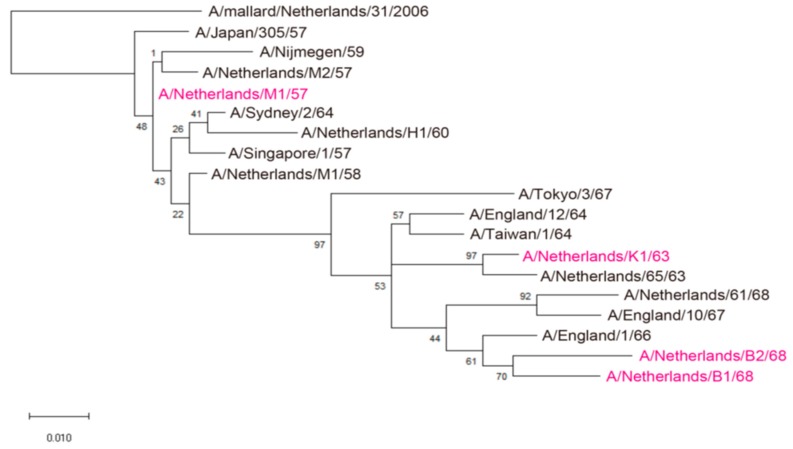
Phylogenetic tree based on the amino acid sequences of HA1 derived from human H2N2 viruses. This tree was constructed by using the Maximum Likelihood method and JTT matrix-based model. Virus isolates used for antigenic analysis are highlighted in magenta.

To examine the antigenicity of the H2N2 viruses that circulated in humans, we used 50 plasma samples that were obtained from aged individuals (Table 2) because individuals who were born between 1928 and 1933 were likely exposed to human H2N2 viruses between 1957 and 1968. We also used 33 plasma samples from younger adults who were born after 1962 (Table 2). We chose 4 human H2N2 isolates—A/Netherlands/M1/57 (H2N2; M1/57), A/Netherlands/K1/63 (H2N2; K1/63), A/Netherlands/B1/68 (H2N2; B1/68), and A/Netherlands/B2/68 (H2N2; B2/68)—based on phylogenic analysis (Figure 1) and a previous report [11]. These isolates were obtained by culture in tertiary monkey kidney cells and MDCK cells for a maximum of five passages, without prior inoculation into embryonated chicken eggs [11].

**Figure 2:**
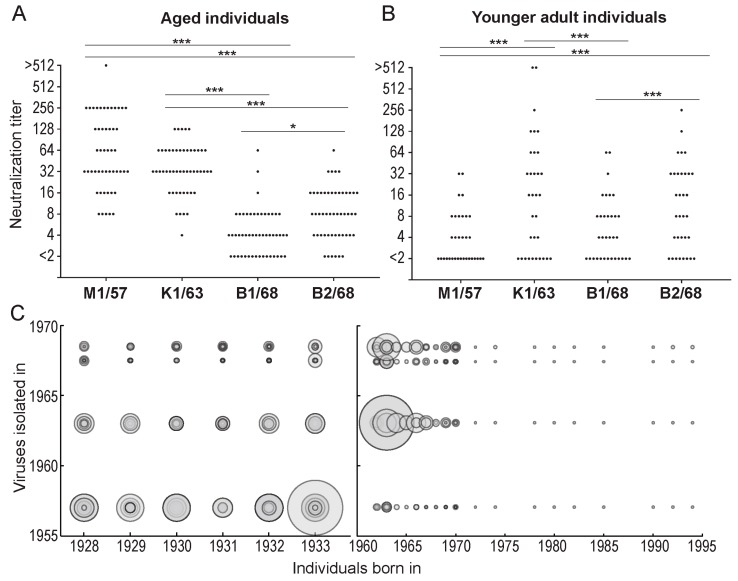
Neutralization titers of aged individuals (**A**) and younger adult individuals (**B**) against human H2N2 viruses. The neutralization titers against the viruses indicated in Table 2 were plotted. A non-parametric Friedman’s test followed by a two-stage linear step-up procedure of Benjamini, Krieger, and Yekutieli was performed. * and *** mean *p* < 0.05 and *p* < 0.0001, respectively. (**C**) Bubble chart of the neutralization titers of all of the individuals. The bigger the circle, the higher the neutralization titer.

**Figure 3:**
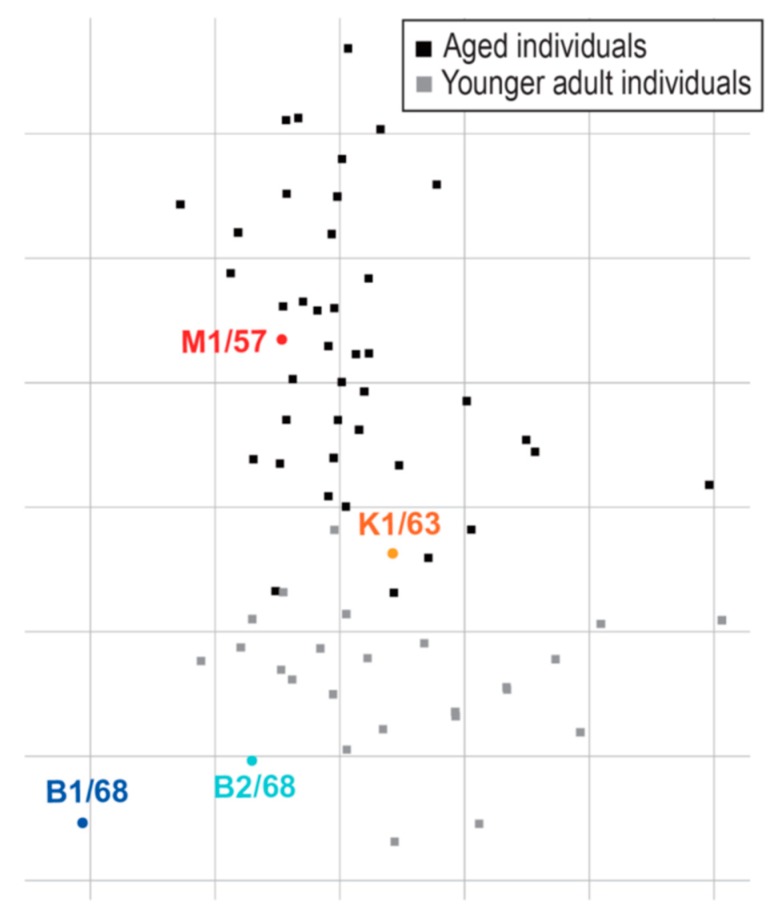
An antigenic map of human H2N2 viruses. An antigenic map was generated from the neutralization data shown in Table 2. Viruses are represented as circles and plasma samples obtained from aged or younger adults are represented as black or grey squares, respectively. Sera with no or only 1 numerical antibody titer are not shown as they cannot be placed properly in an antigenic map. The grid indicates one unit of antigenic distance, a two-fold dilution in neutralization titer.

Using these 83 plasma samples and four human H2N2 isolates, we performed a virus neutralization assay to compare the antigenicity of the human H2N2 isolates (Table 2). The aged individuals (ID #1–50) possessed higher neutralization titers against M1/57 and K1/63 than those against B1/68 and B2/68 (Figure 2A,C). The neutralizing titers against B1/68 of the aged individuals were lower than those against B2/68, whereas those against M1/57 were similar to those against K1/63 (Figure 2A,C). The neutralizing titers against K1/63 and B2/68 of the younger adult individuals were higher than those against M1/57 and B1/68 (Figure 2B,C). Younger adults who were born after 1972 (ID #75–83) had minimal neutralization titers against the human H2N2 viruses tested (Figure 2C). To visualize the antigenicity of the human H2N2 viruses, we constructed an antigenic map using the neutralizing titers of Table 2. This map revealed the antigenic differences among the H2N2 viruses tested and the gradual drift in their antigenicity during circulation in humans (Figure 3). The results obtained with these human sera were similar to those previously obtained using ferret antisera [11].

Human H2N2 viruses circulated in the human population between 1957 and 1968. Mouse and human monoclonal antibodies [9,10] and ferret antisera [6,7,8,11] revealed the antigenic change in H2N2 viruses. Here, we elucidated the antigenic change in H2N2 viruses by using 83 human plasma samples that were obtained from the aged and younger adult individuals. Overall, the antigenicity of H2N2 viruses revealed by human plasma was similar to that revealed by ferret antisera. Ferret antisera showed that the antigenic change between M1/57 and B1/68 was caused by the T128D and R139K substitutions together with other five other changes (Table 3). These substitutions may play a central role in the antigenic change revealed by human plasma since some human neutralizing monoclonal antibodies against H2-HA recognize the region surrounding these seven amino acids [10]. Furthermore, human plasma showed that the antigenicity of B1/68 differed from that of B2/68. At the seven amino acid positions that were important for the antigenic change between M1/57 and B1/68, E126K, P154Q, and A184E substitutions are present between B1/68 and B2/68 (Table 3). These 3 amino acid substitutions may, therefore, contribute to the antigenic difference between the viruses.

Although some of the aged individuals likely have been infected with or exposed to later H2N2 viruses, almost of all of the aged individuals maintained high neutralization titers against the H2N2 viruses that caused the pandemic or its antigenically closely related viruses, but possessed low neutralization titers against later H2N2 viruses. These data suggest that immunological imprinting toward the pandemic H2N2 virus occurred. This would also mean that the immunological imprinting was maintained for 50 years.

Antigenic cartography is usually performed by using antisera obtained from ferrets infected with an influenza virus [13]. In this study, we used human plasma samples obtained from aged and younger adults for the antigenic cartography of human H2N2 viruses. Previous studies showed that human infant sera and ferret antisera see the antigenicity of H1N1 and H3N2 viruses differently [14,15]. However, the positions of four H2N2 viruses in the antigenic map we made with the human plasma samples were similar to those obtained with ferret antisera (Figure 3 and ref [11]). This variation in findings with respect to the antigenic differences recognized by human sera/plasma and ferret antisera between H2N2 and H1N1 or H3N2 is interesting and deserves further study.

Human H2N2 viruses were replaced by human H3N2 viruses in 1968. Therefore, we had thought that people who were born after 1968 were unlikely to possess neutralizing antibodies against human H2N2 viruses. However, they did possess some neutralization titers against the H2N2 viruses. This finding could be explained by antibodies against the HA stem and/or NA because anti-HA stem antibodies are usually cross-reactive with members of group 1 HA [16,17,18] and antibodies against the NA of the seasonal H3N2 virus could suppress H2N2 virus replication, given that the NA of H3N2 virus was inherited from human H2N2 virus [1].

In conclusion, the plasma of individuals who were born between 1928 and 1933 revealed the antigenic change in human H2N2 viruses and the possibility of immunological imprinting of infected individuals against pandemic H2N2 virus, which lasts for half a century.

## Figures and Tables

**Table 1:** Characteristics of the participants in this study.

		No. of Individuals									
		Birth Year									
		Aged Individuals						Younger Adult Individuals			
		1928	1929	1930	1931	1932	1933	1960s	1970s	1980s	1990s
Males		4	4	4	4	4	4	10	5	1	2
Females		5	5	4	4	4	4	9	3	2	1
Total		9	9	8	8	8	8	19	8	3	3
Medical history ^b^	Heart disease	3/9	1/9	3/8	1/8	1/8	1/8	NA ^a^	NA	NA	NA
	Diabetes	0/9	0/9	1/8	2/8	1/8	1/8	NA	NA	NA	NA
	Cancer	3/9	2/9	2/8	4/8	2/8	2/8	NA	NA	NA	NA

^a^ Not available. ^b^ Summary of available medical history of each individual.

**Table 2:** Neutralization titers of aged and younger adult individuals.

ID	Birth Year (age)	M1/57 ^a^	K1/63 ^b^	B1/68 ^c^	B2/68 ^d^
1	1928 (89)	128	8	8	8
2	1928 (89)	8	32	4	16
3	1928 (89)	64	32	<4	16
4	1928 (89)	32	32	4	8
5	1928 (89)	256	128	32	8
6	1928 (89)	64	32	<4	16
7	1928 (89)	256	32	16	16
8	1928 (89)	128	64	4	32
9	1928 (89)	8	16	<4	<4
10	1929 (89)	64	32	8	16
11	1929 (89)	8	32	<4	<4
12	1929 (89)	128	64	4	8
13	1929 (89)	8	32	<4	8
14	1929 (89)	256	64	4	4
15	1929 (89)	32	8	<4	4
16	1929 (89)	32	32	4	<4
17	1929 (88)	32	64	8	8
18	1929 (88)	32	128	4	16
19	1930 (88)	16	16	<4	8
20	1930 (88)	16	8	4	4
21	1930 (88)	32	64	<4	8
22	1930 (88)	256	64	8	4
23	1930 (87)	64	64	4	16
24	1930 (87)	128	32	4	32
25	1930 (87)	256	16	4	16
26	1930 (87)	256	64	8	8
27	1931 (87)	128	64	4	32
28	1931 (87)	16	16	<4	<4
29	1931 (87)	16	64	8	16
30	1931 (87)	32	16	4	16
31	1931 (86)	32	16	<4	<4
32	1931 (86)	32	4	<4	4
33	1931 (86)	32	32	<4	4
34	1931 (86)	128	32	4	8
35	1932 (86)	256	32	8	4
36	1932 (86)	32	32	<4	32
37	1932 (86)	256	32	8	4
38	1932 (86)	16	8	<4	8
39	1932 (86)	256	128	<4	8
40	1932 (85)	256	32	8	16
41	1932 (85)	32	32	<4	4
42	1932 (85)	64	64	4	4
43	1933 (85)	16	16	8	16
44	1933 (85)	256	64	8	8
45	1933 (85)	256	32	4	<4
46	1933 (85)	32	16	4	4
47	1933 (85)	128	64	8	16
48	1933 (85)	>512	128	64	64
49	1933 (85)	64	64	8	16
50	1933 (85)	8	128	4	4
51	1962 (56)	4	64	8	128
52	1962 (55)	<4	16	4	4
53	1962 (55)	8	32	16	32
54	1962 (55)	16	32	8	8
55	1963 (55)	32	>512	32	64
56	1963 (55)	32	>512	64	256
57	1963 (55)	8	128	64	64
58	1963 (55)	16	256	16	32
59	1964 (54)	8	128	8	32
60	1965 (53)	4	64	4	32
61	1966 (52)	8	32	8	32
62	1966 (51)	8	128	16	64
63	1967 (51)	4	64	16	16
64	1967 (50)	<4	32	4	8
65	1968 (50)	<4	4	<4	<4
66	1968 (49)	4	16	4	8
67	1969 (49)	4	32	8	32
68	1969 (49)	<4	<4	<4	<4
69	1969 (48)	4	16	4	16
70	1970 (48)	8	4	8	16
71	1970 (47)	<4	8	4	32
72	1970 (47)	<4	8	<4	16
73	1970 (47)	<4	16	8	4
74	1970 (47)	<4	4	<4	8
75	1972 (45)	<4	<4	<4	<4
76	1974 (44)	<4	<4	<4	4
77	1978 (39)	<4	<4	<4	<4
78	1980 (37)	<4	<4	<4	<4
79	1982 (36)	<4	<4	<4	<4
80	1985 (33)	<4	<4	<4	<4
81	1990 (27)	<4	<4	<4	<4
82	1992 (26)	<4	<4	<4	4
83	1994 (23)	<4	<4	<4	4

Neutralization titers of plasma samples of aged individuals (ID 1–50) and younger adult individuals (ID 51–83) against A/Netherland/M1/57 ^a^, A/Netherland/K1/63 ^b^, A/Netherland/B1/68 ^c^, or A/Netherland/B2/68 ^d^ were measured.

**Table 3:** Comparison of amino acids that are important for HA antigenicity ^a.^

Isolate	Amino Acid Position at						
	126	128	132	139	154	184	188
A/Netherland/M1/57	T	T	R	N	S	T	T
A/Netherland/K1/63	T	T	R	N	P	A	A
A/Netherland/B1/68	E	D	K	K	P	A	A
A/Netherland/B2/68	K	D	K	K	Q	E	A

^a^ Amino acids that are different among the HAs of the four isolates at previously identified antigenic determinants [11] are listed.
